# Nanostructured Materials for Li-Ion Batteries and Beyond

**DOI:** 10.3390/nano6040063

**Published:** 2016-04-07

**Authors:** Xifei Li, Xueliang Sun

**Affiliations:** 1 Energy and Materials Engineering Centre, College of Physics and Materials Science, Tianjin Normal University, Tianjin 300387, China; 2 Tianjin International Joint Research Centre of Surface Technology for Energy Storage Materials, Tianjin 300387, China; 3 Nanomaterials and Energy Lab, Department of Mechanical and Materials Engineering, University of Western Ontario, London, ON N6A 5B9, Canada

This Special Issue “Nanostructured Materials for Li-Ion Batteries and Beyond” of *Nanomaterials* is focused on advancements in the synthesis, optimization, and characterization of nanostructured materials, with an emphasis on the application of nanomaterials for building high performance Li-ion batteries (LIBs) and future systems. The nanostructured materials permit high contact area of the battery’s active materials with its electrolyte and shorten the ion diffusion path, which reveals obvious advantages, increasing the system’s performance. As a result, researchers have continued their dedication in searching for new, facile, low-cost synthetic routes toward novel nanostructured materials and exploring their application in LIBs, Li-S batteries, aqueous batteries, and supercapacitors. The nanostructures employed in this Special Issue include: nanocoatings, nanorods, mesoporous materials and more. An overview of various syntheses and electrochemical performance of these innovative nanostructured materials is further discussed.

High performance Si anodes suffer from large volume change upon cycling in LIBs. Hwang *et al.* [1] reported the structural, mechanical, and electronic properties of graphite-like amorphous carbon coating on bulk silicon to improve the durability of the Si anode using molecular dynamics simulations and ab-initio electronic structure calculations. Chen *et al.* [2] studied Li_4_Ti_5_O_12_ (LTO)/Si composites with different weight ratios. It can be found the electrodes with moderate Si content deliver a stable capacity with good cycling performance, even at a very high current density. The improvement in specific capacity and rate performance was a direct result of the synergy between LTO and Si. Focusing on the cathode, the nanostructured LiCoPO_4_ designed by Manzi and co-workers [3] illustrated the effect of different Co^2+^ sources, solution acidity, and reaction times on the crystal growth of the LiCoPO4 particles by means of a multi-technique approach. Moreover, lithium-excess and nano-sized Li_2+*x*_Mn_1−*x*/2_TiO_4_ (*x* = 0, 0.2, 0.4) cathode materials synthesized via a sol-gel method by Zhang *et al.* [4] revealed that the charge-discharge performance of the nanostructured material was improved remarkably with increasing lithium content.

The design of various morphologies is also presented in this issue. For example, Das *et al.* [5] have studied the numerical simulation of LIBs with the anode made of core-shell heterostructures of silicon-coated carbon nanofibers. Sun *et al.* [6] performed a facile hydrothermal strategy using an H_2_O solvent for the large-scale preparation of finger-like Co_3_O_4_ nanorods. Chang *et al.* [7] demonstrated the application of various sizes of ordered mesoporous carbon nanospheres with diameters of 46–130 nm as active anode materials for LIBs. The facile synthesis of various morphologies might be one of the most promising strategies for high-performance anode materials.

With a low redox potential and a high specific capacity, bismuth was demonstrated as a suitable anode material for aqueous batteries. As part of Zuo and co-workers’ efforts in this area, a bismuth electrode film was directly grown by a facile hydrothermal route and tested in LiOH, NaOH and KOH electrolytes [8]. It was found that, with a smaller *R*_s_ and faster ion diffusion coefficient, a Bi electrode film in KOH electrolyte exhibited better electrochemical performance. Song *et al.* [9] designed a freestanding Si-Ti-Ni (STN) alloy particle/reduced graphene oxide/single wall carbon nanotube composites film. The performance improvements were attributed to the suppression of the pulverization of the STN active material by the excellent mechanical properties of the reduced graphene oxide-single wall carbon nanotube networks and the enhanced kinetics associated with both electron and Li ion transport.

In this issue, special attention is also paid to important systems beyond LIBs. For example, Ye *et al.* [10] found that the introduction of CTAB changes the speciation of S in the Li/S cathode dramatically due to the interaction of CTAB with the terminal S atoms of the polysulfide ions in the Na_2_S*_x_* solution. For the cycled Li/S cell, during the charge/discharge processes, the capacity fade was shown to be due to the loss of electrochemically active sulfur and the accumulation of a compact insulating layer of unexpected sulfur reaction products on the cathode surface. In addition, Liu *et al.* [11] successfully obtained nitrogen-doped banana peel-derived porous carbon as a binder-free electrode for supercapacitors. Their results exhibited a high performance including a high specific surface area of 1357.6 m^2^/g, large pore volume of 0.77 cm^3^/g, a suitable mesopore size distributions around 3.9 nm, and super hydrophilicity due to the nitrogen-containing functional groups.

Due to the advantages of delivering more than one electron, multivalent systems have gained considerable attention. Meanwhile, considering the increasing demand for high energy density batteries and improved safety, in their review article, Guduru and co-workers [12] presented a brief overview of recent progress of multivalent intercalation batteries, including electrode chemistries, functionalities and challenges. Particular attention was paid to Al-ion batteries. Al^3+^ is smaller than Li^+^ in the unsolvated state, but its strongest electrostatic bonding nature with the host electrode materials, similar to many other di- and tri-valent ions, usually results in slower diffusion kinetics within the electrodes. Despite this, it has been found to intercalate/deintercalate into/from certain compounds exhibiting good electrochemical characteristics for battery applications.

In summary, the papers published in this Special Issue provide recent developments of nanostructured active materials in the area of LIBs and future systems. We highly recommend reading this Special Issue, and look forward to your own journal contributions in this fascinating field in the years ahead.
